# Course of IgE to α‐Gal in a Swedish population of α‐Gal syndrome patients

**DOI:** 10.1002/clt2.12087

**Published:** 2021-12-15

**Authors:** Danijela Apostolovic, Jeanette Grundström, Marija Perusko, M. B. Gea Kiewiet, Carl Hamsten, Maria Starkhammar, Marianne van Hage

**Affiliations:** ^1^ Division of Immunology and Allergy, Department of Medicine, Solna Karolinska Institutet and Karolinska University Hospital Stockholm Sweden; ^2^ Faculty of Chemistry, University of Belgrade, Innovation Centre Ltd Belgrade Serbia; ^3^ Department of Internal Medicine Södersjukhuset Stockholm Sweden

1

To the Editor,

The α‐Gal syndrome (AGS) is a severe form of food allergy which has increased worldwide for the past decade. It is caused by IgE antibodies to the carbohydrate galactose‐α‐1,3‐galactose (α‐Gal) found on glycoproteins and glycolipids of non‐primate mammals. Patients develop symptoms after ingestion of mammalian meat and products (e.g. milk, gelatine containing sweets) or pharmaceuticals of mammalian origin (e.g. not fully humanized monoclonal antibodies, antivenoms and vaccines).^1^ A strong relationship between tick bites and the induction of α‐Gal‐specific IgE is a feature specific for AGS^2^, ^3^ as well as the 2–6 h delay in symptoms after mammalian meat consumption. The symptoms range from urticaria, gastro‐intestinal pain or angioedema to severe anaphylaxis. The diagnosis of AGS is complex and is mainly based on the clinical history and IgE reactivity against‐α‐Gal.^4^ There is a need to understand if measuring IgE to α‐Gal over time would be beneficial for the management of AGS patients.

We investigated the IgE levels against α‐Gal and beef (ImmunoCAP System, Thermo Fisher, Uppsala, Sweden) as well as total IgE in 50 well‐characterized Swedish patients with a doctor's diagnosis of AGS^5^ of whom 26 had anaphylaxis (ANA) and 24 non‐anaphylaxis (non‐ANA) (Table S1).

The IgE levels were determined both at the time of diagnosis (visit 1) and at a follow‐up visit (visit 2) at least 3 weeks (median 2.5‐month range <1–102 months) after diagnosis. All patients reported a history of several tick bites at the time of diagnosis.^5^ For 20 patients, visit 2 was more than or equal to 5 months after the diagnosis and included at least one tick season, while for the remaining 30 patients visit 2 was out of the tick season. Among these patients, 18 answered a questionnaire if they had been tick bitten, consuming mammalian meat and milk and dairy products, and experiencing an allergic reaction after the diagnosis. For 17 of these, the IgE levels to tick (*Ixodes ricinus*) at both visits were evaluated as previously.^2^ Among 15/20 patients basophil activation test (BAT) with HSA‐α‐Gal (Dextra Lab, Ltd) was performed as described before, within a positive cut off at ≥ 5% for at least one concentration, including anti/FcεRI.^2^ The study was approved by the Swedish ethical review authority (2011/1604‐31/2, and 2020‐01686) and followed the declaration of Helsinki. Statistical analyses were performed with GraphPad Prism, version 9.1.1 (GraphPad Software). Data are presented as median and range. A *p* value < 0.05 was considered significant.

We found that neither the anti‐α‐Gal IgE levels nor the ratio between anti‐α‐Gal IgE and total IgE correlated with the severity of the allergic reaction (data not shown), which is in line with other studies.^5^, ^6^ When we investigated the fold change of α‐Gal IgE levels between the two visits, we found a significant decrease in the fold change in the non‐ANA group (*n* = 24) compared to the ANA group (*n* = 26) (Figure 1A, *p* = 0.04). We noted that at visit 2, the α‐Gal IgE levels in the ANA group had not changed significantly (Figure 1B, *p* = 0.15), whereas the IgE levels in the non‐ANA group had decreased significantly (Figure 1C, *p* = 0.0008). This was observed regardless of season (winter/summer) for blood collection (Figure S1). There was no difference in time between the visits for the two groups (Figure 1D, *p* = 0.63). The fold change of beef IgE levels were similar between the ANA and non‐ANA groups (*p* = 0.82, data not shown), reflecting that they had been avoiding mammalian meat to the same extent. In the group of patients with visit 2 ≤5 months after the diagnosis, we observed that in 20/30 the IgE levels decreased (Table S1), probably due to the lack of new tick bites. However, when only patients with the visit 2 ≥ 5 months after the diagnosis were selected, the IgE levels to α‐Gal decreased in the non‐ANA group, but not significantly (Figure S2A, *p* = 0.07). Likewise, the IgE levels to beef decreased significantly in the non‐ANA group only (Figure S2B, *p* = 0.016).

**FIGURE 1:**
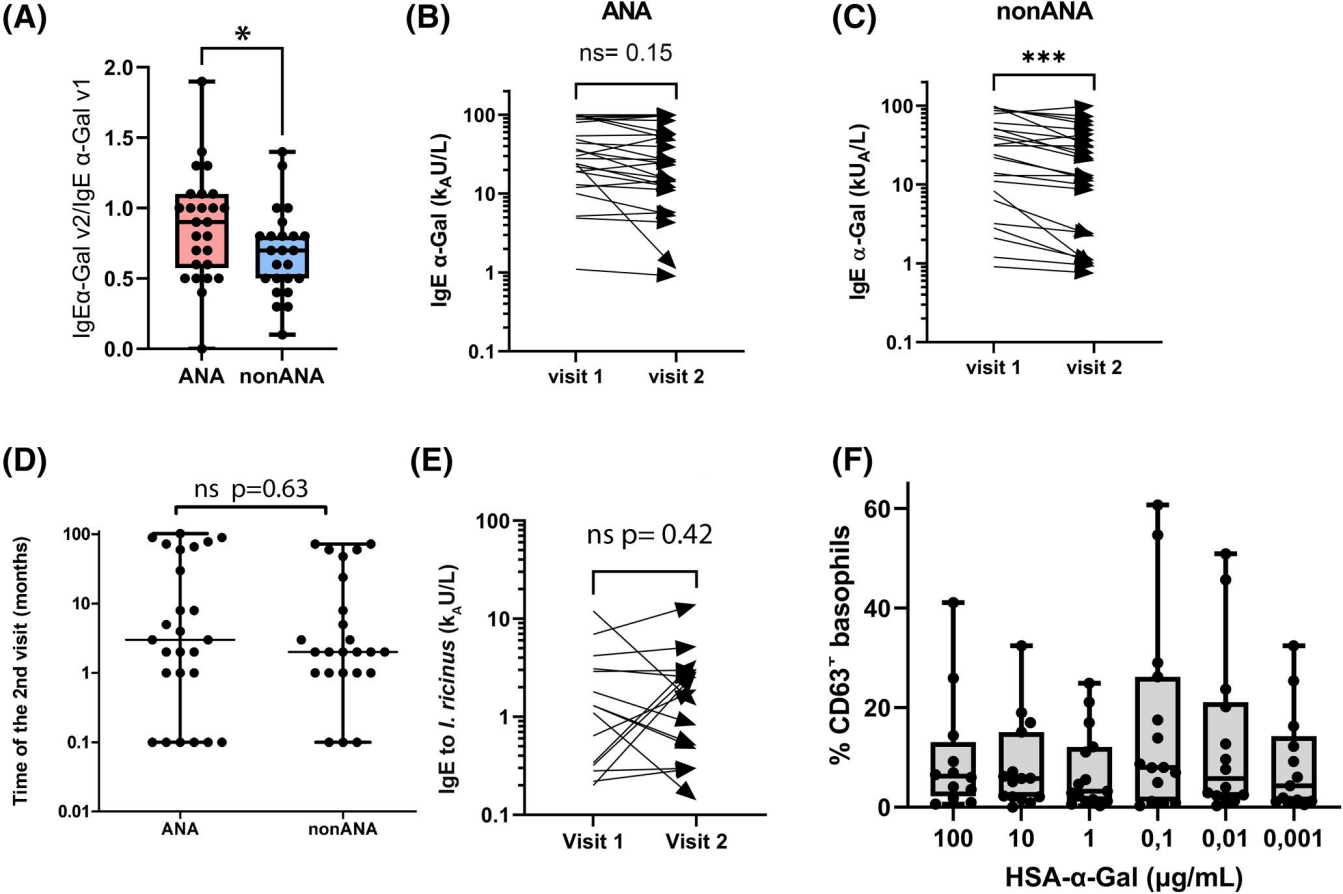
IgE and basophil reactivity among AGS patients at visit 1 and visit 2 (≥5 months after the diagnosis). (A) IgE fold change against α‐Gal over time between anaphylactic (ANA, *n* = 26) and non‐anaphylactic (non‐ANA, *n* = 24) patients. (B) IgE levels to α‐Gal at both visits in ANA and (C) non‐ANA patients. (D) Comparison of time differences between ANA (*n* = 26) and non‐ANA patients (*n* = 24). (E) IgE levels to *I. ricinus* (*n* = 17); (F) Basophil reactivity against different concentrations of HSA‐α‐Gal (*n* = 15). Unpaired Mann–Whitney and Wilcoxon matched‐pairs test between groups were used for comparison. *p < 0.05; ***p < 0.001

When the patients whose visit 2 was ≥5 months after the diagnosis (18/20) were interviewed, all except one had avoided mammalian foods, and 17/18 reported consuming milk and dairy products. The remaining patient (No.44) reported consuming meat 2–5 times per year. The majority (15/18) had experienced symptoms after diagnosis, 72% (13/18) gastro‐intestinal discomfort, and 55.6% (10/18) urticaria, probably due to non‐awareness of consuming α‐Gal‐containing food. Only three patients (16.6%) reported an absence of allergic symptoms. With respect to tick bites, 72% of the interviewed patients (13/18) reported that they had been tick bitten after the diagnosis (Table S1). Their IgE levels to tick did not change significantly between visits (Figure 1E, *p* = 0.42). Of five patients who did not report tick bites, three had at least a three‐fold decrease in α‐Gal IgE levels (No. 38, No. 41, and No. 46). Moreover, one of them had not experienced symptoms after the diagnosis (No. 38). Our IgE data are firmly in line with previous reports showing that anti‐α‐Gal and anti‐tick IgE antibodies are maintained by continuous tick bites,^7^ and that avoiding tick bites leads to decreased anti‐α‐Gal and total IgE levels throughout time.^8^

Furthermore, 15 patients with a second visit ≥5 months after the diagnosis were tested in BAT against HSA‐α‐Gal (ANA *n* = 10, non‐ANA *n* = 5). Basophils from 10 patients were activated at different concentrations of HSA‐α‐Gal (Figure 1F), showing a atypical dose‐dependent reactivity patterns. However, there was no difference in basophil reactivity between ANA and non‐ANA patients. In two of five BAT‐negative patients (No. 39 and No. 46) IgE levels to α‐Gal decreased, and only one (No. 43) had not reported allergic symptoms after AGS diagnosis. For the remaining 2 BAT‐negative (No. 33 and No. 35), neither their allergen‐specific IgE levels nor their symptoms could explain the lack of basophil reactivity. This disconcordance has been previously reported with food allergens.^9^

In conclusion, we found that the IgE levels to α‐Gal in AGS patients experiencing anaphylaxis are maintained over time. Although the patients avoided mammalian meat, most had been tick bitten and experienced symptoms, probably caused by the consumption of α‐Gal‐containing food. This is in concordance with still having basophils that are activated by α‐Gal. Thus, AGS patients with severe reactions should strictly be avoiding α‐Gal containing food as well as tick bites.

## CONFLICT OF INTEREST

Prof. M. van Hage reports personal fees from Thermo Fisher Scientific, outside the submitted work. Dr. M. Starkhammar reports personal fees from ALK, personal fees from CHIESI, personal fees from MEDA, personal fees from AstraZeneca, outside the submitted work. The rest of the authors have nothing to disclose.

## Supporting information

Table S1

Figure S1

Figure S2
