# Supplementary material for: Course of IgE to α‐Gal in a Swedish population of α‐Gal syndrome patients
Source: Clin Transl Allergy. 2021 Dec 15;11(10):e12087. doi: 10.1002/clt2.12087 (PMC8672165; doi:10.1002/clt2.12087)
Supplement: Supplementary file 1 — Table S1 [file CLT2-11-e12087-s001.docx]

Supporting Information

**Course of IgE to α-Gal in a Swedish population of α-Gal syndrome patients**

Authors: **Danijela Apostolovic, PhD^1^, Jeanette Grundström, PhD^1^, Marija Perusko PhD^1,2^, M. B. Gea Kiewiet PhD^1^, Carl Hamsten PhD^1^, Maria Starkhammar, MD^3^, Marianne van Hage, MD PhD^1*^**

^1^ Department of Medicine Solna, Division of Immunology and Allergy, Karolinska Institutet and Karolinska University Hospital, Stockholm, Sweden;

^2^ Innovative Centre Faculty of Chemistry, Belgrade, Serbia;

^3^ Department of Internal Medicine, Södersjukhuset, Stockholm, Sweden;

* Correspondences:

Marianne van Hage, MD PhD

Visionsgatan 4, NKS BioClinicum J7:30

Immunology and Allergy Division, Department of Medicine

Karolinska Institutet

17164

Stockholm

Sweden

E-mail: marianne.van.hage@ki.se

**Table S1 - Serological characteristics of AGS allergic patients**

| ID | Symptoms (ANA/non-ANA) | | | α-Gal IgE  (kU_A_/l) | | Beef IgE  (kU_A_/l) | | | | *I.ricinus* IgE  (kU_A_/l) | Total IgE (kU/l) | α-Gal IgE  (kU_A_/l) | | | Beef IgE  (kU_A_/l) | | | *I. ricinus* IgE  (kU_A_/l) | | Time between visits  (months) | | |  | | Reported tick bite within a year before visit 2 | | Reported mammalian meat consumption after AGS diagnosis | | | | Reported symptoms after AGS diagnosis | | Performed  BAT | |  |
| --- | --- | --- | --- | --- | --- | --- | --- | --- | --- | --- | --- | --- | --- | --- | --- | --- | --- | --- | --- | --- | --- | --- | --- | --- | --- | --- | --- | --- | --- | --- | --- | --- | --- | --- | --- |
|  | Visit 1 | | | | | | | | | | | | |  | | |  | |  | | | Visit 2 | | | | | | | | | | | | |  |
| 1 | ANA | | | 4.9 | 1.3 | | | | 0.22 | | 18 | 4.3 | | | 1.4 | | | nd | | | <1 | |  | | | | |  | |  | |  | |  | |
| 2 | non-ANA | | | 13.0 | 1.2 | | | | 0.13 | | 44 | 13.0 | | | nd | | | nd | | | <1 | |  | | | | |  | |  | |  | |  | |
| 3 | non-ANA | | | 93.0 | 13 | | | | 4.10 | | 240 | 63.0 | | | 16 | | | nd | | | <1 | |  | | | | |  | |  | |  | |  | |
| 4 | ANA | | | 13.0 | 3.8 | | | | 2.90 | | 48 | 11.0 | | | nd | | | nd | | | <1 | |  | | | | |  | |  | |  | |  | |
| 5 | ANA | | | 22.0 | 6 | | | | 0.28 | | 110 | 15.0 | | | 1.4 | | | nd | | | <1 | |  | | | | |  | |  | |  | |  | |
| 6 | non-ANA | | | 3.2 | 0.6 | | | | <0.1 | | 72 | 2.4 | | | 0.6 | | | nd | | | <1 | |  | | | | |  | |  | |  | |  | |
| 7 | ANA | | | 28.0 | 12 | | | | 2.20 | | 150 | 27.0 | | | 11 | | | nd | | | <1 | |  | | | | |  | |  | |  | |  | |
| 8 | ANA | | | 80.0 | 16 | | | | 3.40 | | 310 | >100 | | | 54 | | | nd | | | <1 | |  | | | | |  | |  | |  | |  | |
| 9 | ANA | | | 1.1 | 0.61 | | | | <0.1 | | 110 | 0.9 | | | 0.46 | | | nd | | | <1 | |  | | | | |  | |  | |  | |  | |
| 10 | non-ANA | | | 31.0 | 8.4 | | | | 2.30 | | 80 | 31.0 | | | 11 | | | nd | | | 1 | |  | | | | |  | |  | |  | |  | |
| 11 | ANA | | | 5.2 | 2.1 | | | | <0.1 | | 47 | 5.8 | | | 1.2 | | | nd | | | 1 | |  | | | | |  | |  | |  | |  | |
| 12 | non-ANA | | | 0.9 | 0.39 | | | | <0.1 | | 71 | 0.8 | | | 0.5 | | | nd | | | 1 | |  | | | | |  | |  | |  | |  | |
| 13 | ANA | | | 44.0 | 15 | | | | 2.30 | | 310 | 39.0 | | | 3.2 | | | nd | | | 1 | |  | | | | |  | |  | |  | |  | |
| 14 | non-ANA | | | 1.2 | 0.7 | | | | <0.1 | | 28 | 0.9 | | | 0.5 | | | nd | | | 1 | |  | | | | |  | |  | |  | |  | |
| 15 | non-ANA | | | 32.0 | 8.4 | | | | 0.79 | | 440 | 44.0 | | | 8.4 | | | nd | | | 1 | |  | | | | |  | |  | |  | |  | |
| 16 | non-ANA | | | 50.0 | 31 | | | | 21.00 | | 410 | 36.0 | | | 28 | | | nd | | | 1 | |  | | | | |  | |  | |  | |  | |
| 17 | ANA | | | 94.0 | 9.7 | | | | 0.30 | | 270 | >100 | | | 30 | | | nd | | | 1 | |  | | | | |  | |  | |  | |  | |
| 18 | non-ANA | | | 42.2 | 5.7 | | | | 0.45 | | 207 | 25.0 | | | 5.9 | | | nd | | | 2 | |  | | | | |  | |  | |  | |  | |
| 19 | non-ANA | | | 95.2 | 32.5 | | | | 2.30 | | 1576 | 56.0 | | | 22 | | | nd | | | 2 | |  | | | | |  | |  | |  | |  | |
| 20 | non-ANA | | | 87.0 | 8.9 | | | | 1.40 | | 217 | 73.0 | | | 6.3 | | | nd | | | 2 | |  | | | | |  | |  | |  | |  | |
| 21 | non-ANA | | | 11.0 | 2.6 | | | | 0.20 | | 45 | 8.5 | | | 2.1 | | | nd | | | 2 | |  | | | | |  | |  | |  | |  | |
| 22 | ANA | | | >100 | 26 | | | | 7.80 | | 320 | 47.0 | | | 29 | | | nd | | | 2 | |  | | | | |  | |  | |  | |  | |
| 23 | ANA | | | 95.0 | 25 | | | | 7.30 | | 300 | 99.0 | | | 32 | | | nd | | | 2 | |  | | | | |  | |  | |  | |  | |
| 24 | non-ANA | | | 14.0 | 4.3 | | | | 0.39 | | 130 | 9.6 | | | 2.7 | | | nd | | | 2 | |  | | | | |  | |  | |  | |  | |
| 25 | ANA | | | 35.0 | 13 | | | | 3.60 | | 150 | 23.0 | | | 12 | | | nd | | | 2 | |  | | | | |  | |  | |  | |  | |
| 26 | non-ANA | | | 2.1 | 0.3 | | | | <0.1 | | 28 | 1.1 | | | 0.2 | | | nd | | | 3 | |  | | | | |  | |  | |  | |  | |
| 27 | non-ANA | | | 40.0 | 1.4 | | | | 0.36 | | 830 | 20.0 | | | 1.6 | | | nd | | | 3 | |  | | | | |  | |  | |  | |  | |
| 28 | ANA | | | 10.0 | 3.9 | | | | 0.93 | | 37 | 5.2 | | | 2.4 | | | nd | | | 3 | |  | | | | |  | |  | |  | |  | |
| 29 | ANA | | | >100 | 47 | | | | 3.50 | | 420 | >100 | | | 25 | | | nd | | | 3 | |  | | | | |  | |  | |  | |  | |
| 30 | ANA | | | 49.0 | 29 | | | | 6.50 | | 190 | 25.0 | | | 18 | | | nd | | | 4 | |  | | | | |  | |  | |  | |  | |
| 31 | ANA | | | 88.0 | 34 | | | | 12.0 | | 2100 | >100 | | | 13 | | | 1.3 | | | 5 | | nd | | | | | nd | | nd | | YES | |  | |
| 32 | non-ANA | | | 24.0 | 7.9 | | | | 1.30 | | 81 | 11.0 | | | 4.9 | | | 0.51 | | | 5 | | YES | | | | | NO | | GI | | YES | |  | |
| 33 | ANA | | | 30.0 | 17 | | | | 0.2 | | 140 | 57 | | | 25 | | | 2.9 | | | 8 | | NO | | | | | NO | | U | | YES | |  | |
| 34 | non-ANA | | | 52.0 | 14 | | | | <0.1 | | 60 | 21 | | | 4.9 | | | 0.32 | | | 8 | | YES | | | | | NO | | NO SYMPTOMS | | YES | |  | |
| 35 | ANA | | | 54.0 | 19 | | | | 4.20 | | 450 | 56.0 | | | 29 | | | 5.2 | | | 18 | | YES | | | | | NO | | GI, U | | YES | |  | |
| 36 | non-ANA | | | 79.0 | 18 | | | | 0.34 | | 2000 | >100 | | | nd | | | 3.80 | | | 24 | | nd | | | | | nd | | nd | | YES | |  | |
| 37 | ANA | | | 24.0 | 6.8 | | | | 0.22 | | 86 | 12.0 | | | 3.8 | | | 0.3 | | | 30 | | YES | | | | | NO | | GI, U | | YES | |  | |
| 38 | non-ANA | | | 8.3 | 1.6 | | | | <0.1 | | 65 | 0.9 | | | 0.68 | | | <0.10 | | | 48 | | NO | | | | | NO | | NO SYMPTOMS | | NO | |  | |
| 39 | non-ANA | | | 6.3 | 1.9 | | | | nd | | 155 | 2.2 | | | 0.7 | | | 0.27 | | | 60 | | YES | | | | | NO | | GI | | YES | |  | |
| 40 | ANA | | | 19.0 | 2.6 | | | | 0.28 | | 67 | 12.0 | | | 3.2 | | | 0.30 | | | 60 | | YES | | | | | NO | | GI | | NO | |  | |
| 41 | non-ANA | | | 2.8 | 0.6 | | | | 0.64 | | 817 | 1.0 | | | 0.38 | | | 1.90 | | | 60 | | NO | | | | | NO | | GI, U | | NO | |  | |
| 42 | non-ANA | | | 99.0 | 62 | | | | 1.30 | | 320 | 29.0 | | | 19 | | | 0.47 | | | 60 | | YES | | | | | NO | | GI | | NO | |  | |
| 43 | ANA | | | >100 | 60.5 | | | | 6.93 | | 840 | 57.0 | | | 28 | | | 14.0 | | | 66 | | YES | | | | | NO | | NO SYMPTOMS | | YES | |  | |
| 44 | non-ANA | | | 22.0 | 1.9 | | | | nd | | 140 | 12.0 | | | 1.6 | | | 1.20 | | | 72 | | YES | | | | | YES | | GI, U | | YES | |  | |
| 45 | non-ANA | | | 61.0 | 12 | | | | 1.80 | | 360 | 49.0 | | | 11 | | | 0.82 | | | 72 | | YES | | | | | NO | | GI, U | | N0 | |  | |
| 46 | ANA | | | 24.0 | 8.7 | | | | 1.10 | | 150 | 1.1 | | | 0.8 | | | 0.14 | | | 72 | | NO† | | | | | NO | | GI, U | | YES | |  | |
| 47 | ANA | | | 37.0 | 9.9 | | | | 0.32 | | 98 | 14.0 | | | 6.6 | | | 3.10 | | | 78 | | YES | | | | | NO | | GI, U | | YES | |  | |
| 48 | ANA | | | 12.0 | 2.8 | | | | 1.20 | | 110 | 15.0 | | | nd | | | nd | | | 90 | | NO | | | | | NO | | GI | | YES | |  | |
| 49 | ANA | | | 88.0 | 11.4 | | | | 3.10 | | 390 | 84.0 | | | 22 | | | 2.50 | | | 90 | | YES | | | | | NO | | GI, U | | YES | |  | |
| 50 | ANA | | | 19.0 | 3.8 | | | | 2.90 | | 140 | 26.0 | | | 14 | | | 3.0 | | | 102 | | YES | | | | | NO | | U | | YES | |  | |
| Median | | |  | 30.5 | 8.4 | | | | 1.01 | | 150 | 22.0 | | | 6.3 | | | 1.01 | | | 2.5 | |  | | | | |  |  | |  | | |  | |
| Range | |  | | 0.9- >100 | | | 0.3-62 | <0.1- 21 | | | 18 - 2100 |  | 0.8 - >100 | | | 0.2-54 | | <0.1 - 14 | | <1 - 102 | | | |  | |  | | |  | |  | | | |  |

ANA-anaphylaxis; non-ANA - non-anaphylaxis; nd – not determined; † - bitten after diagnosis but not in last 2 years; GI – gastro-intestinal; U – urticaria.

Anaphylaxis was defined a severe, usually rapid onset and potentially life-threatening systemic hypersensitivity reaction from several organ systems, which always includes respiratory and/or cardiovascular manifestations. Symptoms from the skin, mucous membranes, gastrointestinal tract, and the central nervous system are usually seen as stated in Kiewiet MBG, et al. JACI in Practice 2020, 8(6):2027-2034.e
