# Supplementary figures and images for: Course of IgE to α‐Gal in a Swedish population of α‐Gal syndrome patients
Source: Clin Transl Allergy. 2021 Dec 15;11(10):e12087. doi: 10.1002/clt2.12087 (PMC8672165; doi:10.1002/clt2.12087)

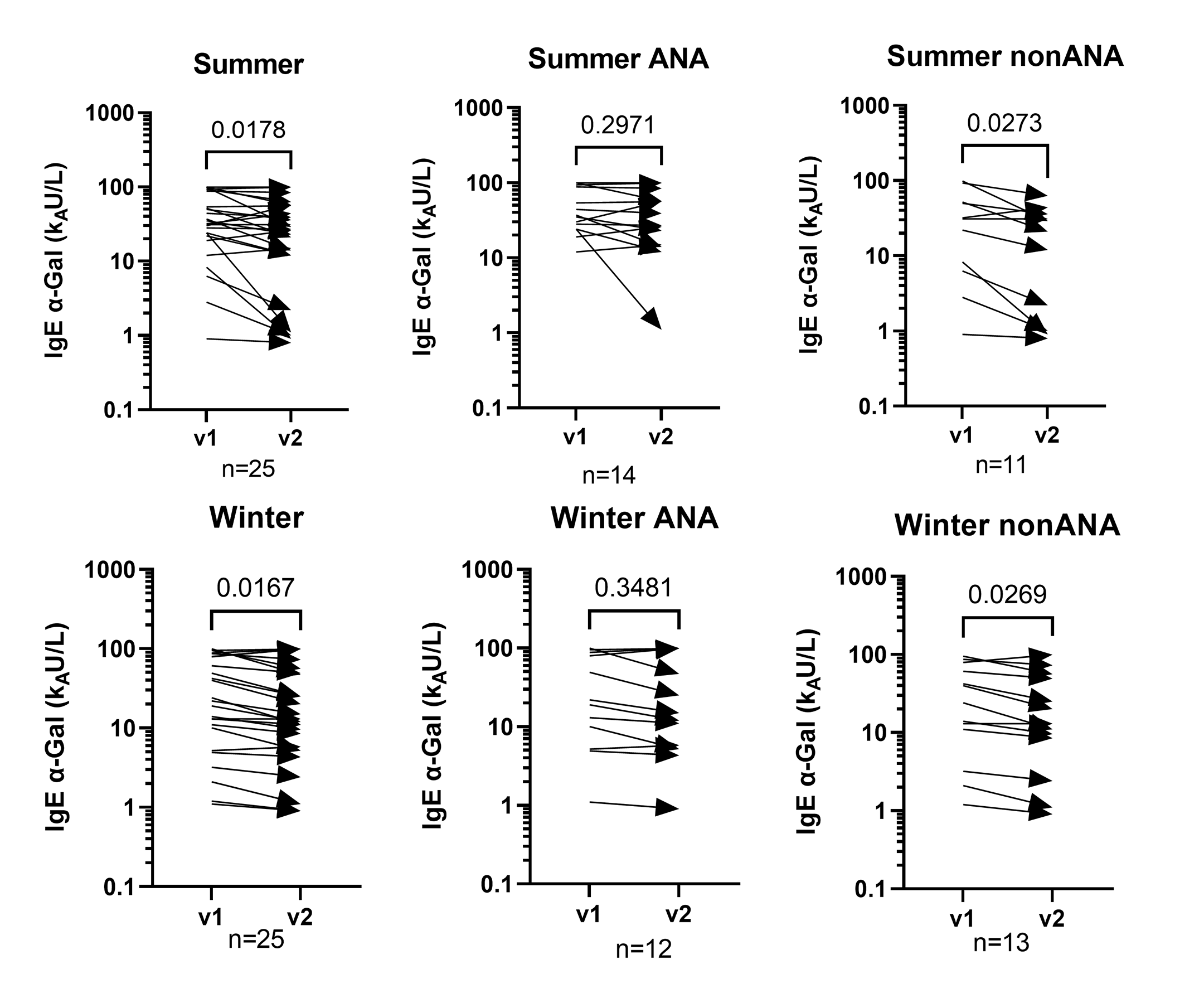

Supplement: Supplementary file 2 — Figure S1 [file CLT2-11-e12087-s002.tif]

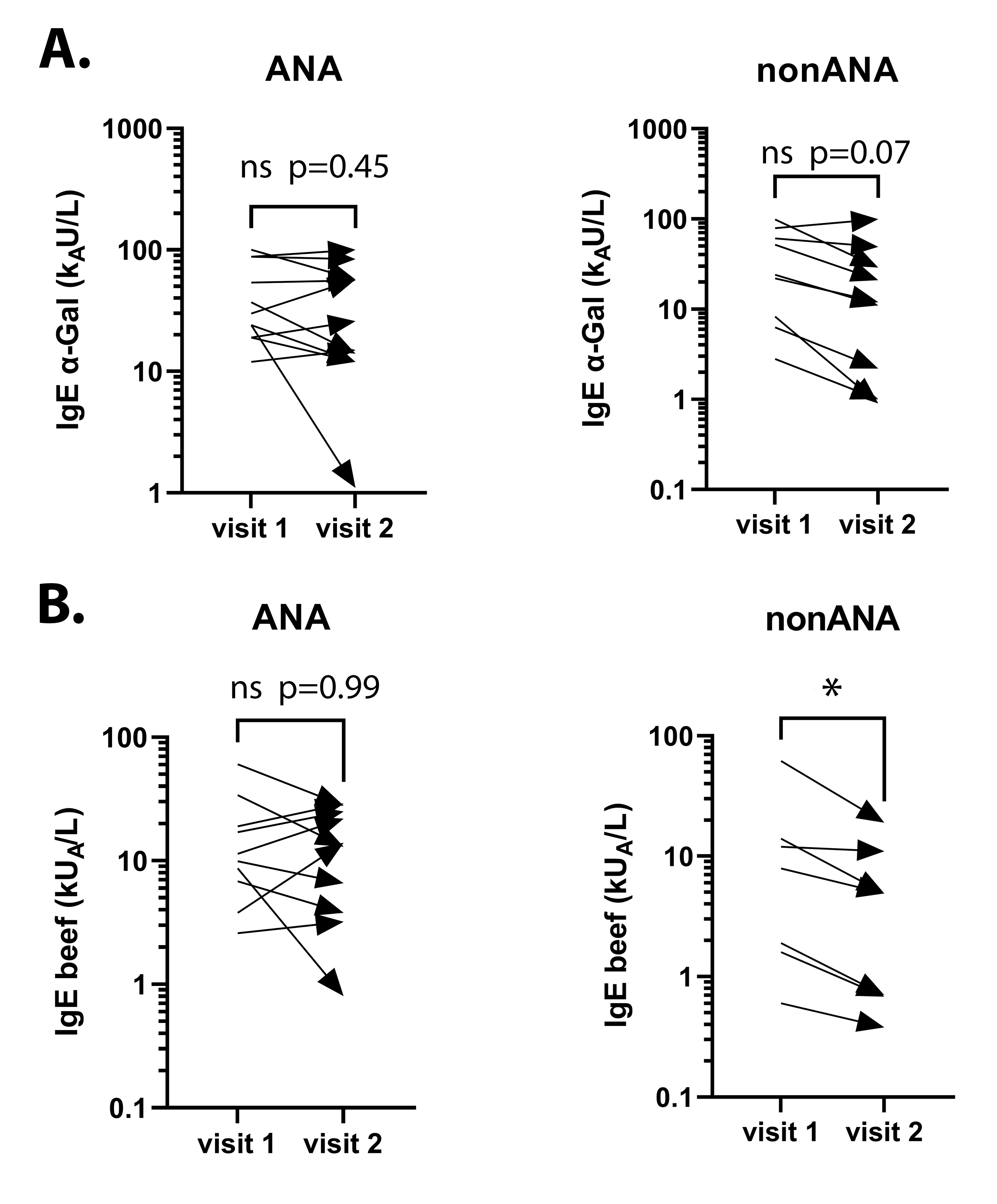

Supplement: Supplementary file 3 — Figure S2 [file CLT2-11-e12087-s003.tif]
